# Whose Responsibility Is It? Implementing Patient-Prioritized Healthcare System Change in Oncology

**DOI:** 10.3390/curroncol31110538

**Published:** 2024-11-18

**Authors:** Holly Etchegary, John King, Sevtap Savas

**Affiliations:** 1 Public Interest Group on Cancer Research, St. John’s, NL A1B 3V6, Canada; johnking6@gmail.com (J.K.); savas@mun.ca (S.S.); 2 Division of Population Health and Applied Health Sciences, Faculty of Medicine, Memorial University, St. John’s, NL A1B 3V6, Canada; 3 Patient/Public Author, St. John’s, NL A5A 4L8, Canada; 4 Division of Biomedical Sciences, Faculty of Medicine, Memorial University, St. John’s, NL A1B 3V6, Canada; 5 Discipline of Oncology, Faculty of Medicine, Memorial University, St. John’s, NL A1B 3V6, Canada

**Keywords:** patient engagement, patient centered, patient oriented research, knowledge translation, cancer care, oncology

## Abstract

This brief commentary describes the reflections on a fundamental question by the Public Interest Group on Cancer Research, a successful academic-community partnership focused on cancer research, education, public engagement, and advocacy in Canada’s Eastern province of Newfoundland and Labrador. Our Group has achieved some success in a short time with very limited funding. It has successfully created public spaces for conversations about cancer care and priorities for research and regularly advocated for health service change prioritized by input from patients and family members. However, we remain challenged in our understanding of how to truly implement change within oncology care contexts that is informed by patients and families affected by cancer. In this short reflection, we hope to raise awareness of this important issue and question whose responsibility it is to work with patients and families and follow through on prioritized healthcare issues and services. We suggest this may be a matter of integrated knowledge translation and a better understanding of where patients and families fit in this space. We hope to encourage reflection and conversation among all relevant stakeholders about how best to implement patient-prioritized change in oncology care and policy.

## 1. Background and Context

The Public Interest Group on Cancer Research (PI Group) is a successful academic-community partnership focused on cancer research, education, public engagement, and advocacy [1,2]. The Group brought together scientists, health system representatives, and patients and family members affected by cancer in 2021 with a mission to bring “impactful and positive change to the lives of individuals affected by cancer” [2].

The founding members of the Group included a cancer scientist, a health and social scientist, an oncologist, an oncology health system administrator, and two community members with lived experience of cancer [1]. Founding members co-created a recruitment strategy for additional patient and family members, successfully recruiting 12 public members in the first year of the Group. These members represented a diversity of lived cancer experiences, ages, professional backgrounds, and geographic regions of the province. All group members attend meetings, suggest agenda items, co-develop and deliver group initiatives and activities, and assist with public outreach. While the Group’s membership has evolved due to changing personal and professional circumstances, patient and family member involvement remains strong, with more limited participation of health system members. Currently, the Group includes three scientists, with one representing the largest health service zone in the province, and nine patients and family members with lived experience of cancer.

Since its inception, the PI Group has co-created patient-oriented research and public engagement project applications for funding, organized a free public conference on cancer, and regularly advocated on issues identified by patients and family members through social and traditional media [2]. More recently, its activities include a dedicated podcast in an effort to continue exchanging knowledge and conversations about cancer (listen here: https://open.spotify.com/show/0dty5nEXGPNOCpp6l3t11I (accessed on 10 November 2024), as well as targeted public conversations (e.g., the lived experience of transgender individuals’ cancer care).

The Group’s core is situated at Memorial University, the only university in the Eastern Canadian province of Newfoundland and Labrador (NL). According to yearly Canadian cancer statistics, residents of NL consistently have some of the highest age-standardized cancer risks and mortality rates in Canada (e.g., [3,4]). Thus, cancer is highly relevant to the NL public and provincial healthcare system.

Locally, the university [5] and the province [6] have a strong commitment to patient and public engagement, in line with Canada’s Strategy for Patient-Oriented Research [7,8] and global calls for engaging with citizens in the design and delivery of healthcare and health policy-making (e.g., [9,10]). Thus, the PI Group’s values, mission, and activities fit well into a national and global culture of patient and public engagement in oncology care and research.

## 2. A Key Question—Whose Responsibility Is It?

The PI Group has demonstrated success at raising awareness about cancer screening, local cancer care services, and lived experiences, [1,2] designing research and public engagement projects (e.g., [11]) and creating community spaces where patients, the general public, and oncology professionals can come together [12,13]. Our Group defines success through a number of metrics, including an annual members’ survey to assess satisfaction, uptake, and attendance at public events we organize, the number of new initiatives we undertake, and traditional outputs such as conference presentations and scholarly papers. However, implementing patient and family-prioritized change in oncology care remains a challenge. Indeed, in our local cancer care context, our Group has not made significant progress in translating the recommendations from patients and family members into the provision of cancer care services or policy. Yet, within oncology, patient engagement has long been recognized as a key element of high-quality care (e.g., [14]).

Patient and public partnership roles in health research are now largely common (and particularly so in oncology [9,10,11,12,13,14,15]) but comparatively less so in the realms of health policy and governance, health technology assessment, and health education (e.g., see ref. [9] for a recent systematic review). While more common in health planning/service design and quality improvement, even here, patient partner activities and patients’ fit within organizational structures and decision-making processes are often only vaguely described [9].

Our Group has been reflecting on our place and role within our local oncology care context. Specifically, we have been discussing ‘whose responsibility is it?’ to respond to patient and family concerns about their cancer care and health system experiences. Who exactly is responsible for implementing changes to healthcare services and healthcare policy that are informed by patient and family engagement? Whose job is it to inform, listen to, and work with patients and families affected by cancer?

Upon quick consideration, the answer seems obvious—the healthcare system. If challenges and gaps are identified along the cancer care journey, then wouldn’t those responsible for delivering that care be obvious stakeholders to respond to such challenges? But this answer is too simplistic and somewhat nebulous. What do we mean by ‘the healthcare system’? Who in the healthcare system? A complaints department? Oncology program managers? Cancer Centre directors? Oncologists? Other oncology providers? Hospital directors? Others? Some combination of all these? Where do patient and family priorities and suggestions even fit within the myriad competing priorities of healthcare systems? For example, locally [16,17] and across Canada [18], healthcare system recruitment is currently a major challenge and a priority for health systems and governments.

What role do governments play? Given that healthcare is decentralized in Canada, do provincial governments bear any responsibility to allocate funding or create programs within cancer care services that specifically address patient and family-identified priorities? Like healthcare systems, governments also have multiple competing priorities, so it is difficult to understand just how public and patient engagement fits into decision-making.

How about academic institutions? Many cancer researchers and scientists, as well as health providers with faculty appointments, are affiliated with academic medical centers. This is certainly the case in our local context. What is their role in trying to implement patient-oriented research and public engagement findings into practice? Do they have any role? And if so, what is it?

And what about Groups like ours? And other advocates, such as charitable organizations and patient advocacy networks? Here, we mean those individuals and groups—like ours—with a mission to create positive change in the lives of patients and families affected by cancer. How can we help to generate change within local healthcare systems and services beyond continuously advocating? Or is that the most important, or even sole, role of groups such as ours?

## 3. Challenges with Moving Findings into Practice

We wonder if this is fundamentally a question of integrated knowledge translation (IKT) and implementation, complex processes with many challenges to the implementation of findings into practice [19,20,21]. Effectively implementing patient-centered change into oncology healthcare provision and service planning likely requires collaboration across and input from all relevant groups and organizations. This seems logical and very easy to say. But, our experience suggests it is very difficult to accomplish in practice.

As Carlson and colleagues noted, relevant and related areas of clinical care, research, and education/training “…usually operate in silos, with different budgets, different administration, different funding sources, and formulas, different policies, different personnel, different priorities, completely different buildings, etc.…-rendering translation of research results into everyday practice prohibitive.” [22] (p. 2). In response, they describe a program that successfully integrated integrative oncology interventions and approaches into a provincial, publicly funded cancer care agency in Canada [22]. The ACTION Centre is a formal structure and a collaborative partnership between the University of Calgary and the provincial health authority in Alberta responsible for the delivery of cancer care [22]. It provides one example of a model to support knowledge translation and implementation into cancer care. While there are many examples of patient engagement in cancer research projects, [9] we are less aware of real-world examples such as these situated within provincial clinical care contexts. The ACTION Centre’s Steering Committee is the decision-making body, and half of its members are patients [22]. This structure certainly seems to ‘walk the walk’, so to speak, giving patients a voice in decision-making.

Within the context of our Group, there are specific challenges to implementing patient-driven changes to local oncology care and services. Currently, Group membership includes only one health system representative, signifying a gap in key perspectives such as oncologists, other frontline oncology staff, and health policy decision-makers in cancer care. Without the direct participation of (and collaboration with) high-level oncology decision-makers, it will likely remain a challenge to prioritize patient and family suggestions for cancer care. While our Group has been very successful in receiving funding for engagement and advocacy initiatives (e.g., a free public conference on cancer and a dedicated podcast), we have been unsuccessful in patient-oriented research funding applications that aim to explore research questions prioritized by patients and family members. One could argue that had these been funded, moving findings into local practice would have been facilitated, given the involvement of health system stakeholders in applications.

That said, we do not believe one-off grant funding is not the best way to promote sustainable change in healthcare systems. All too often, if academic teams are lucky enough to find funding for a patient-oriented project, the findings and recommendations for care fall by the wayside when the project (i.e., funding) ends.

## 4. Where Do Patients and Families Fit?

Patients and family members are very well suited to make meaningful contributions to integrated knowledge translation teams and research [23]. As a knowledge user, they are significantly impacted by oncology research and clinical care decisions, and as such, their perspectives are inherently valuable. However, the ways in which patients and family members have been incorporated into IKT teams and the nature and scope of their engagement are underreported or non-existent [23]. The IKT literature suggests that most knowledge users in IKT teams to date have been those providing frontline services or those with decision-making authority in a healthcare organization, not patients and family members [23]. Thus, there is room for improvement in the inclusion of this key group of stakeholders in IKT generally, and we would suggest oncology specifically.

We fully agree that IKT approaches with representative perspectives from all relevant stakeholders (including patients and families) likely have the best chance of making meaningful changes and creating positive health outcomes for patients [22,23]. However, we are stymied as to how to identify and engage champions within the healthcare system, local governments, and medical academic centers to prioritize those projects—among multiple other competing priorities—that will ultimately get implemented into practice and create positive health outcomes for patients and families. Our Group regularly invites key stakeholders to relevant engagement initiatives and research applications, as well as provides annual reports of patient and family member priorities and our Group’s activities and learnings to oncology care providers and cancer care decision-makers. While this knowledge exchange is important (and we hope, valued), we are generally unaware of how oncology program and service decisions are ultimately taken and exactly how patient and family member engagement fits into these [24].

## 5. Moving Forward: Better Integration of Patient and Family Voices in Oncology Care and Policy

Our Group hopes to raise awareness of this important issue and contribute to the discussion.

We have recently advocated for the creation of a province-wide engagement network to better encourage collaborative partnerships among all stakeholders, including our Group, and the breakdown of traditional siloed ways of working [11]. If we are to truly make oncology care and policy decisions that are informed by patients and families, we must first create opportunities for them to sit at decision-making tables. These opportunities should ideally be ongoing and championed by health system decision-makers. Environmental scans and systematic reviews of how and when patients and families have been engaged and their impact on decisions in the context of oncology healthcare would be useful for identifying successful examples and lessons learned.

Better transparency is needed regarding how oncology health service provision and policy decisions are typically made. Until we understand exactly how these decisions are made and exactly who is making them, it is difficult to understand whether and how the voice of patients and families fits in the process. The assessment of patient-reported outcome and experience measures (PROMs and PREMs, respectively) offers one systematic way to integrate patient voices into the delivery of cancer care [25,26,27]. However, while their use in clinical research has grown, assessment in routine care delivery is far less common [26,27]. In the cancer care context, transparency about who makes the decision to systematically collect these kinds of patient data is also needed.

Finally, considerations of power are also relevant here. Traditionally, patients and families have been regarded as passive recipients of healthcare, while power has largely remained in the hands of healthcare providers and health system funders [28,29]. Without acknowledging this power imbalance and the ways in which power differentials affect the relationships among patients, caregivers, providers, and decision-makers, advancing truly patient-centered and informed care and policy in oncology will remain challenging. Decision makers are encouraged to rebalance power through the creation of ‘invited spaces’ [28], such as forums where patient and family members sit alongside high-level health system decision-makers and providers and contribute to decision-making discussions. Ultimately, organizational culture change is required in health system policy development—one that recognizes and respects patient perspectives—to address power imbalances that prevent true patient and family engagement [29].

## 6. Conclusions

While significant strides have been made in the inclusion of patient partners in research settings, there remains room for improvement in their inclusion in health system and policy decisions. Here, we have described some of the work of the Public Interest Group on Cancer Research and raised questions about whose responsibility it is to facilitate better integration of patient partnership in cancer care service and policy decisions. Specifically, our Group has been reflecting on how and where patient and family voices fit among multiple competing priorities of health systems and policy processes. We believe the questions raised in this commentary are useful for stakeholders involved in oncology care and policy to consider. Ultimately, we aim to encourage reflection on these issues and support the transparent inclusion of patient and family voices in health system decisions in oncology care.
